# Development and initial validation of a palliative care readiness (PALCARE) tool for older adults with cancer: Study protocol

**DOI:** 10.1371/journal.pone.0323366

**Published:** 2025-05-21

**Authors:** Jyotsana Parajuli, Zhuo Job Chen, Regine M. Smith, Liliana M. Hernandez, Savannah Norris, Sarah Rutledge, Grant Williams, Marie Bakitas, Simon Hsiang

**Affiliations:** 1 University of North Carolina at Charlotte, Charlotte, North Carolina, United States of America; 2 Atrium Health, Charlotte, North Carolina, United States of America; 3 University of Alabama at Birmingham, Birmingham, Alabama, United States of America; PLOS: Public Library of Science, UNITED KINGDOM OF GREAT BRITAIN AND NORTHERN IRELAND

## Abstract

**Introduction:**

Early palliative care (PC) has been recommended for older adults with cancer to address their cancer and treatment related high symptom burden and unmet needs. However, it is underutilized; older adults with cancer are 60% less likely to use PC compared to their younger counterparts. One approach to reduce existing barriers and improve utilization is to assess their readiness for PC. However, there are no gold-standard tools to measure older adults with cancer PC readiness.

**Methods:**

Informed by the 8 domains of PC recommended by National Consensus Project guidelines, Readiness to Change Framework, and principles of community engaged research, we will use a mixed methods approach, to develop and validate a Palliative Care Readiness (PALCARE) Tool through the following steps: 1) Identify items using semi-structured interviews with 20 diverse (race/ethnicity, rural/urban residence, cancer types) older adults with cancer; 2) Develop draft tool via expert panel surveys followed by focus group with PC experts, and 3) Determine item clarity and wording via cognitive interviews with 20 dyads of older adults with cancer and their family caregivers.

**Discussion/Conclusion:**

The PALCARE tool will be one of the first measures of PC readiness among a diverse sample of older adults with cancer. Once validated, PALCARE can enhance clinical practice by identifying those who are/are not ready for PC, providing targeted PC that is congruent with their readiness, and educating and supporting those who report being not yet ready for PC. The ultimate goal of this study is to improve PC early access and older adults’ quality of life.

## Introduction

Cancer is the second most common cause of death in the United States (US) affecting more than 18 million individuals [1]. Cancer incidence greatly increases with age; approximately 60% of cancer diagnoses and 70% of all cancer-related deaths occur in adults aged 65 and over [2]. Older adults with cancer experience multiple physical and psychological symptoms (e.g., pain, nausea, vomiting, diarrhea, anorexia, weight loss, malnutrition, fatigue, depression, insomnia) [3–5] and high levels of unmet physical, psychological, informational, communication, and sexual health needs [6–7] which significantly affects their quality of life (QoL). Concurrently, the aging process creates bio-psycho-social changes resulting in health and support needs [8] and is associated with occurrence of multiple chronic conditions that may worsen with cancer-related toxicities [9,10].

High symptom burden, high level of unmet needs, and presence of multiple chronic conditions increase the complexity of cancer care and cancer related decision-making in older adults with cancer. Therefore, they can greatly benefit from palliative care (PC). Early PC, typically defined as integration of PC within 8–12 weeks of diagnosis [11–13] can lead to positive outcomes such as symptom relief; improved mood, QoL and survival; increased illness understanding; overall satisfaction with care and treatment outcomes; reduced depressive symptoms; lower ICU admissions and length of stay; and reduced costs of care [11–18]. Professional organizations such as the World Health Organization [19], American Society of Clinical Oncology [20], National Consensus Project’s Clinical Practice Guidelines for Quality Palliative Care [21], and National Comprehensive Cancer Network [22] have responded to the growing needs of adults with cancer and recommend integrating PC into standard oncology care to provide quality holistic care to patients with cancer and their families [23].

Despite the positive benefits, PC is often not a part of routine cancer care among older adults with cancer and is introduced late in the disease trajectory. Many adults with cancer receive PC in the last four weeks of life, when earlier intervention would have been beneficial [24]. Older adults may show different triggers for the initiation of PC than younger populations such as frailty, pre-existing functional dependence, cognitive impairment, emotional distress, and caregiver-related problems [25]. However, older adults are 60% less likely to utilize PC compared to their younger counterparts [26,27]. PC barriers include lack of knowledge and awareness of PC, negative stereotypes/stigma (e.g., associating PC with hopelessness, death, forgoing cancer therapy, and end-of-life care) [28,29], denying the terminal nature of the disease, and requesting not to be referred to PC or not showing up to PC clinics or missing PC appointments when referred [30,31]. Other characteristics associated with low PC use are being male, unmarried, a racial minority, low socio-economic status, residing in a rural area, and fee-for-service enrollees [32]. Lack of or delays in PC referral result in poor symptom management, reduced QoL, and unwanted uncomfortable end-of-life care [33].

One approach to reducing barriers and improving PC utilization among older adults with cancer is to systematically measure their readiness for PC. The concept of ‘readiness’ has been established in previous studies as integral to making and maintaining a health behavioral change including for excessive drinking [34], smoking [35], substance abuse [36], exercise [37], and adherence to treatment [38]. Evidence shows that when patients experience a high degree of readiness, they view their disease condition in a more positive light and report less anger and depression [39]. Readiness also has been studied in the context of advance care planning [40] but not in the context of PC among patients with cancer.

There is no standardized tool to measure readiness for early PC in older adults with cancer. While there are tools to decide the timing for PC referral, most tools focus on providers’ perspectives on triggers for PC referrals [41–48] and do not focus specifically on patients with cancer [40–52]. One tool [53] examines perceptions of – but not readiness for PC and is not tailored for older adults. Most tools address just one or two NCP guidelines domains [50]; none cover all eight domains of PC [41–53]. Currently, one tool “Advance Care Planning Engagement Survey” assesses readiness in advance care planning decision-making [40] but focuses on end-of-life decision-making rather than early PC. Finally, one PC instrument [53], “Perceptions of Palliative Care Instrument (PPCI)” includes one global question (domain) on readiness to hear about PC, namely “*How ready are you to hear about palliative care today?”* (Response options, 0 = not at all to 10 = totally). This question, however, targets the readiness to hear about PC -- not readiness for accepting or receiving it. It does not ask about readiness for PC in the eight specific domains of PC listed in the NCP guidelines, and it is not specifically designed for older adults with cancer [53]. There is an urgent need to develop a PC readiness tool that holistically assesses all eight NCP domains of PC and is specifically tailored to meet the needs of older adults with cancer. The proposed Palliative Care Readiness (PALCARE) tool will expand the range of PC domains by including all eight domains of PC listed in the NCP guidelines and will be guided by the Readiness to Change Framework. A tool assessing PC readiness can allow the oncology team to provide targeted PC in the domains where they feel ready, educate and suggest benefits in domains where they are not yet ready, and ultimately prepare them to accept referral to interdisciplinary PC team which may lead to patients’ improved quality of life.

## Guiding Frameworks

Our study is guided by a synthesized theoretical framework using a) National Consensus Project (NCP) for Quality Palliative Care guidelines [21] and b) Readiness to Change Framework [39].

### NCP Project Guidelines

The NCP project created clinical practice guidelines targeted towards improving PC quality and standards across all care settings [21]. The NCP guidelines describe eight domains of quality PC: 1) structure and process of care; 2) physical aspects of care; 3) psychological and psychiatric aspects of care; 4) social aspects of care; 5) spiritual, religious, and existential aspects of care; 6) cultural aspects of care; 7) end-of-life care; 8) ethical and legal aspects of care [21] which can ensure holistic and equitable quality PC to older adults with cancer.

### Readiness to Change Framework

Dalton and Gotlieb [39] developed the Readiness to Change Framework which states three phases that characterize the process of readiness prior to a behavioral change: a) realizing what needs to change, b) weighing the cost and benefit, and c) planning for action. The first phase is becoming aware of and realizing what needs to change. The second phase involves appraising the costs and benefits of the proposed change and determining whether to change or not. When an individual decides that the benefits of the proposed change are greater than the costs, the final phase of the readiness process begins. The final readiness phase involves their desire or willingness to change and planning for action towards that change [39]. Using this framework, the readiness for PC among older adults with cancer may occur in three phases: 1) realizing they have high symptom burden and other unmet needs that may benefit from PC; 2) weighing PC costs and benefits of PC; and 3) planning to receive PC.

**Fig 1:**
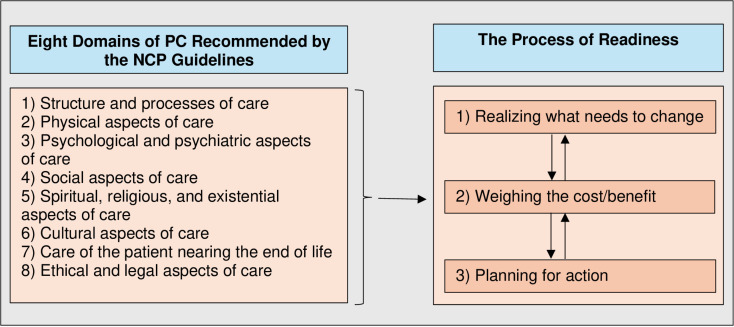
Conceptual Framework.

Fig 1 illustrates the eight domains of PC recommended by the NCP guidelines that PALCARE items will cover and the three phases of readiness that will be used to construct response categories for the questions.

### Principles of community engaged research

This research will be informed by the principles of community engaged research [54] Community engagement is a process where researchers work collaboratively with groups of people to identify and address issues that are affecting their health/well-being. The aim of community engaged research is to build trust, identify new resources, create better communication between researchers and community members, and create long-standing collaborations [54]. Community engaged research has been shown to address health disparities resulting in positive health outcomes [54–56]. Community based participatory approach (CBPR) is considered to be the gold standard to reduce disparities, create and administer culturally concordant palliative care interventions, and improve health outcomes among individuals with serious illness [56–58].

For this R15 research project, we will use the ‘continuum of community engagement in research’ framework developed/recommended by Key and colleagues [54] which can range from consultation with community partners to community driven research [54]. They have identified 7 levels of community engagement in research: 1) No community involvement 2) Community informed 3) Community Consultation 4) Community Participation 5) Community Initiated 6) Community Based Participatory Research 7) Community Driven/Community Led [54]. For this research, we will use the third level of community engagement, ‘*community consultation’* by forming a patient and family advisory board consisting of older adults with cancer and their family caregivers to seek feedback on study flyers, other recruitment materials, and semi-structured interview guide questions. Evidence shows that collaborating on interview guide with community members can allow researchers to seek feedback on question depth, appropriateness, and clarity and ensure that the questions asked are more representative of the population (especially those from underrepresented backgrounds) [59].

The PALCARE tool is proposed to be developed and validated for diverse older adults with cancer comprising Caucasian/White, African American/Black, and Hispanic race/ethnicity. Therefore, we will assemble a patient and family advisory board of approximately 6–8 members representative of the above race/ethnicities and seek feedback on study materials (*which has been formed and convened as of December 2024*)*.*

## Materials and methods

### Study design

Using a mixed methods approach, this 3- year study will iteratively develop and establish the content validity and preliminary convergent validity and test-retest reliability of the PALCARE tool for older adults with cancer through three specific aims.

#### Aim 1.

Develop the Palliative Care Readiness tool (PALCARE) for older adults with cancer. To develop the initial items pool, semi-structured interviews will be conducted with a diverse group of 20 older adults with cancer (diverse race/ethnicity, rural/urban residence, common types of cancer).

#### Aim 2.

Establish the content validity of the PALCARE tool. We will assess content validity in three steps: a) two rounds of expert panel review (via an online survey) with 10 PC experts including physicians, nurses, social workers, and chaplains; b) a focus group with 6 PC experts; c) cognitive interviews with 20 dyads of older adults with cancer and their family caregivers (40 in total) to gain additional insights on PALCARE questions.

#### Aim 3.

Establish preliminary convergent validity and test-retest reliability of the PALCARE tool: We will assess a) convergent validity of the PALCARE tool by correlating the PALCARE with the global readiness to hear about PC from the Perceptions of Palliative Care Instrument (PPCI) [53], and b) test-retest reliability by assessing readiness for PC using the PALCARE tool during the cognitive interview and 2 weeks afterward.

The three specific aims which will be conducted over 4 phases (see Table 1).

**Table 1:** Overview of methods/specific aim for each study phase.

Specific Aims	Study phase
1) Develop PALCARE tool	**Phase 1**: Semi-structured interviews with a diverse group of 20 older adults with cancer (White, Black, and Hispanics)
2) Establish the content validity of the PALCARE tool	**Phase 2**: Two rounds of expert panel survey with 10 PC experts; **Phase 3**: Focus group with 6 PC experts **Phase 4**: Cognitive interviews with 20 diverse group of dyads of older adults with cancer and their family caregivers
3) Establish the preliminary convergent validity and test-retest reliability of the PALCARE tool	**Phase 4**: Cognitive interviews with measurement of the global question on PC readiness from the Perceptions of Palliative Care Instrument (PPCI) and repeatedly assessing PALCARE before the cognitive interview and 2 weeks afterward.

## Phase 1: Semi-structured interviews

We will develop the initial items pool via semi-structured interviews with a diverse group of 20 older adults with cancer (diverse race/ethnicity, rural/urban residence, common types of cancer). This sample size was chosen based on previous studies who recruited 15–20 participants for initial development of PC tools [53,60]. We anticipate that 20 participants will provide data saturation, but we will continue to recruit until data saturation [61]. Semi-structured interviews will be led by the study PI at times and locations that are convenient for the participants. Institutional Review Board approval for this protocol has been obtained from the University of North Carolina at Charlotte and Atrium Health (recruitment site).

### Eligibility criteria.

Participant eligibility will include the following: a) ≥60 years, b) diagnosis of advanced cancer (metastatic, stage III or IV solid tumors), c) no current enrollment in hospice care; d) ability to communicate in English. Exclusion criteria include a) those under age 60 years, b) non-advanced cancer, c) enrolled in hospice care, d) inability to communicate in English, and e) cognitive impairment as indicated by the Callahan assessment tool [62]. We selected the age criteria of 60 years and above for recruitment because recent evidence suggests that geriatric impairments and frailty are prevalent and commonly occur in individuals as early as age 60 years [63]. Also, cancer occurs in much earlier age among patients belonging to racial/ethnic minority backgrounds [64].

### Recruitment.

Participants will be recruited from a cancer center located in North Carolina. We aim to purposively and proportionately recruit older adults with cancer from diverse racial and ethnic backgrounds (White, Black, Hispanic) from rural and urban areas and diagnosed with one of the four most common cancers in older adults (colorectal, lung and bronchus, prostate, breast). Although preferences will be given to the four most common cancers, we will include older adults with any advanced cancer. Participants will be recruited by our health systems collaborator by using electronic health records (EHR) to identify eligible study participants. To further enhance recruitment, we will also employ a multi-method approach including posted flyers, personal visits to the cancer center, and referrals from other clinicians and key stakeholders.

### Data collection.

Informed consent will be obtained from participants prior to data collection (written informed consent for in-person interviews and verbal consents for virtual participants). After completing informed consent, interested and eligible older adults with cancer will be asked to complete the demographic health form that will consist of participant’s age, sex, ethnicity, race, educational level, marital status, employment status, annual household income, health insurance, cancer type and stage, and place of residence (rural vs urban). We will use semi-structured interviews to generate initial PALCARE items. The research team has developed a semi-structured interview guide which will include real-life scenarios related to PC readiness and will identify factors related to the eight domains of PC listed in the NCP guidelines (See Table 2 for general topics for each of the 8 domains). We will begin the interview by asking participants an overarching question “Tell me about a common symptom/situation you have experienced where you needed care or support.” Directed probe questions/statements will be used to identify readiness for PC in each eight domains of PC listed in the NCP guidelines. Participants may choose to receive a $50 Amazon gift card to thank them for their time. Data collection is planned to begin from late March of 2025. We anticipate having a first draft of the PALCARE tool by December of 2025.

**Table 2:** Example Topics for Item Generation for all 8 Domains of PC Listed in the NCP Guidelines.

Eight Domains of PC	Example of Topics
1. Structure and process of care	Conversations with the patient, family caregivers, and clinicians; patient and family understanding of the serious illness; goals of care; treatment preferences;
2. Physical aspects of care	Pain and other physical symptoms; functional status; symptom distress; QoL.
3. Psychological and psychiatric aspects of care	Emotional distress; anxiety; depression; delirium; dementia; family dynamics; coping strategies.
4. Social aspects of care	Family structure and function; quality of relationships; communication; decision-making preferences; social support; resiliency; support system for respite; community resources;
5. Spiritual, religious, and existential aspects of care	Sources of spiritual strength and support; existential concerns such as lack of meaning; question of existence; meaning and suffering; relationship with God;
6. Cultural aspects of care	Cultural practices, beliefs, values during serious illness; dying process; time of death and post death; preferred names and pronouns; interaction with the PC team; truth telling or not sharing diagnosis; preferred taboo and practices; health literacy; acculturation.
7. Care of the patient nearing the end of life	End of life: signs and symptoms that patient is nearing death; prepare caregivers how to recognize and manage common symptoms; document patients’ wishes and preferences;
8. Ethical and legal aspects of care	Advance directives; patients’ values and preferences; spiritual beliefs are routinely reviewed and documented with attention to changes in health care status

### Data analysis.

All qualitative interview data will be audio-taped, transcribed verbatim, and checked for accuracy. Data will be analyzed using content analysis [65] aided by NVivo software. Two researchers will code each transcript. Dr. Parajuli will lead the analysis. Content analysis will lead to generation of a list of codes which will be reviewed for redundancy. An initial item pool will then be developed from the codes with items in each of the 8 domains listed in the NCP guidelines.

### Phase 2: Expert panel review to establish the content validity of the PALCARE tool

To establish content validity, two rounds of expert panel review will be conducted with 10 PC and geriatric oncology experts who will independently review the PALCARE tool. These diverse experts will include four physicians, two nurses, two social workers, and two chaplains. Using an iterative process, experts will independently score each PALCARE item to see if it assesses PC readiness and addresses each of the eight PC domains. This step is essential in determining whether the content is pertinent to PC theory and practice and will help refine the tool as necessary.

#### Recruitment.

Experts will be recruited from the research team’s professional networks: PC and geriatric oncology related organizations such as Hospice and Palliative Nurses Association (HPNA), International Society of Geriatric Oncology (SIOG), and Cancer and Aging Research Group (CARG), based on their clinical experiences and expertise working with older adults with cancer from diverse racial and ethnic backgrounds, types of cancer, rural/urban residence with the target population [66] (such as certification or full-time employment for at least three years in a setting caring for older adults with cancer and PC).

#### Data collection.

Each expert will be invited to participate in the expert panel review with a standardized explanation of the study. They will meet with the PI if they have questions. After they provide informed consent, we will send experts a Qualtrics online survey which will describe the goal of the tool, including the initial item pool. Experts will be asked to initially evaluate each item on a visual analogue Likert-type scale ranging from 1 to 10 (1 = extremely inadequate to 10 = extremely adequate) based on the following criteria a) relevance of each item to PC readiness, b) how well each item reflects the respective eight PC domains listed in the NCP guidelines, c) readability and appropriateness of the item for older adults with cancer. For any item the expert rate ≤ 6 (out of 10), a comment box will be provided where they can address each item’s relevance, readability, and appropriateness. Items will remain if they receive an average rating of greater than 6. Finally, panelists will be asked to recommend items that may have been missed. Based on their recommendations, items will be revised, dropped, or added. We will then send the revised item pool to the same experts for a second round of review. Each survey is expected to take approximately 30 minutes.

#### Data analysis.

After data collection is complete, information will be cleaned and stored on the private and secure computers of the research team. Responses will be reviewed to further refine the item pool. The study team will review descriptive and qualitative feedback to identify problematic items that need revision. The content validity index (CVI) will determine the relevance of items, evidence of the validity of the content [67] and retention of items in the tool [68]. At the end of the second expert panel review, items with an CVI < 0.78 will be considered problematic and will be deleted or modified.

### Phase 3: A focus group discussion with the PC experts

Clinical voices of PC experts and nuances of the readiness concept may not be fully explored in an online survey. Therefore, a one-time focus group [69] will be conducted with an expert panel to obtain additional insights on the important aspects of the PC content and its relevance to older adults with cancer. We will also incorporate clinician/health system barriers into the discussion during this focus group session. Further, we will gather perspectives from these experienced physicians, nurses, social workers, and chaplains who have observed how patients evolve in their understanding of PC. Finally, we will gather information on what interventions regarding readiness for PC would be useful in clinical settings to gather ideas for future intervention development after all the psychometric properties of this readiness tool have been established.

#### Recruitment.

The same group of experts who participated in the online survey will be invited to participate in the focus group discussion with a sample goal of 6 participant informants.

#### Data collection.

After obtaining the informed consent, the focus group discussion will be moderated by the PI using a semi-structured interview guide and is anticipated to last for approximately 60 minutes. The research team will develop clarifying and open questions regarding each item on the initial PALCARE tool from the online expert panel review survey. The focus group will be conducted virtually and audio recorded. The audio files will be transcribed and fully de-identified before analysis.

#### Data analysis.

After the focus group, the moderator will debrief with other members of the research team to identify and note initial impressions and critical points of the conversation. Data will be analyzed using focus group methodology that includes a) familiarization, b) indexing, c) charting, d) mapping, and e) interpretation [69].

### Phase 4: Cognitive interviews with older adults with cancer and their family caregivers

Cognitive interviews will be conducted with 20 dyads of older adults with cancer and their family caregivers. The NCP guidelines [54] have emphasized that family caregivers are an integral part of palliative and hospice care services alongside patients with serious illnesses. Especially for older adults with cancer, family caregivers can provide information about their health and function. They also can help older adults better understand PC. It, therefore, is essential that family caregivers be involved in understanding readiness for PC in older adults with cancer [70]. Caregivers will include self-endorsing or identified by the patient as an unpaid spouse/partner, relative, or friend who has a close relationship with them, assists them in their medical care, and does not have to live in the same household [71]. To get individual perspectives from the caregivers, we will conduct cognitive interviews separately with the older adults with cancer and their caregivers. Including family caregivers’ perspectives will help us understand the congruence/incongruence about readiness for PC and inform not only the development of the readiness tool but also the administration of the tool (timing, mode of delivery) and future interventions.

Cognitive interviews help researchers develop items for an instrument that are appropriate, comprehensive, and easily understandable by the participants in the target population [72]. Cognitive interviewing uses a combination of verbal probing and think-aloud alone techniques [73]. This process helps researchers understand participant’s decisions by examining the thought processes behind their responses. Verbal probing allows for clarification and comments on wording of question items [74]. Think-aloud technique helps researchers understand what kind of information the participants remember while answering a question [73]. Cognitive interviews improve the validity and reliability of a survey in two ways: (a) provide additional evidence on items that address salient aspects of participants’ experiences and further strengthen the content validity; (b) help refine ambiguously or poorly worded items and enhance questionnaire reliability [73].

#### Recruitment.

Cognitive interviews will be conducted in 20 older adults with cancer and 20 family caregivers. Participants will be purposively selected to reflect a diversity of cancer types, race/ethnicity (White, Black, Hispanic), and rural versus urban residence. Criteria for family caregivers include a) Being at least 18 years of age; b) Identifying themselves as the family caregiver or being identified as such by the older adult with cancer; c) Ability to communicate in English. Each participant will receive a $50 Amazon gift card as a thank you for completing the survey.

#### Data collection.

After obtaining informed consent, PALCARE items will be evaluated using cognitive interviewing techniques with a sample of 20 dyads of older adults with cancer and their family caregivers. The substantial sample size of n = 40 for the cognitive interviews will enable us to identify potential problems with the questions and maximally fine-tune the tool [75]. Verbal probing approach of cognitive interviews will be used to solicit the participants’ understanding and interpretation of each item in the PALCARE tool. Verbal probing will allow participants to ask for clarification and comments on wording of each item in the PALCARE tool. We will randomly order the items in the PALCARE tool to ensure the eight PC domains are dispersed throughout the questionnaire [72]. During the cognitive interviewing process, the interviewer will read each question to the participants and ask that they verbalize their understanding of each question. The cognitive interviews will be conducted using a structured interview guide according to the established standard guidelines for cognitive interviewing [74]. According to the guidelines, the cognitive interview will consist of the following process: (a) a PALCARE item will be asked, and the respondent will respond; (b) a series of verbal probes will be asked in reference to that PALCARE item and the respondent’s answer; (c) this process will be repeated for each PALCARE item. Probes will be developed to evaluate several features relevant to item structure and respondent decisional processes according to the verbal probing guidelines provided by Willis, 2005 [74] and will be applied consistently across all PALCARE items. All interviews will be audio recorded.

To help establish preliminary a) convergent validity of the PALCARE tool with the global question on PC readiness from the Perceptions of Palliative Care Instrument (PPCI) [53], and b) test-retest reliability by repeatedly assessing PALCARE before the cognitive interview and 2 weeks afterward, we will ask participants to a) rate their PC readiness using the one question from the PPCI instrument “How ready are you to hear about palliative care today?” (Scoring option, 0 = not at all to 10 = totally); and b) rate their PC readiness using our PALCARE tool before the cognitive interview and 2 weeks after the cognitive interview.

#### Data analysis.

Audio recordings of the cognitive interviews will be transcribed and fully de-identified. Several procedures will be followed to ensure rigor and adherence to the cognitive interview procedure and appropriate verbal probing. Each researcher will review results to make decisions on revising, retaining, deleting, or adding items in the PALCARE tool. As recommended by previous literature, we will review each item to examine participants’ understanding and ability to respond to each item. Correlations of the PALCARE and the global readiness will be used to demonstrate convergent validity. Correlations of the two assessments of PALCARE will be used to demonstrate test-retest reliability. In addition, we will compare the scores across pretest and posttest using paired-samples t-tests.

The Question Appraisal System (QAS) [76] will be used to analyze the interview data and identify sources of response bias within and across the PALCARE items. The QAS is a coding tool used to evaluate questions based on prespecified types of problems within categories. The QAS has eight problem categories: a) reading; b) instructions; c) clarity; d) assumptions; e) knowledge/memory; f) sensitivity bias; g) response categories; and h) other. The first QAS category targets researchers and is not applicable to older adults with cancer. Thus, we will examine each PALCARE item for the seven remaining QAS problem categories. Each category has multiple specific problem types that could be identified [75]. Decisions of retaining, revising, or deleting items will depend on its importance or relevance to PC readiness in older adults with cancer, clarity of instructions to complete the survey, difficulty of items etc. The eight problem categories of QAS and their description are listed in Table 3.

**Table 3:** QAS Overall Categories and Their Description.

QAS Category	Description
1. Reading	Determine if it is difficult for the interviewers to read the question uniformly to all respondents.
2. Instructions	Look for problems with any introductions, instructions, or explanations from the respondent’s point of view.
3. Clarity	Identify problems to communicating the intent or meaning of the question to the respondent.
4. Assumptions	Determine if there are problems with assumptions made or the underlying logic.
5. Knowledge/memory	Check whether respondents are likely to not know or have trouble remembering information.
6. Sensitivity/bias	Assess questions for sensitive nature or wording, and for bias.
7. Response categories	Assess the adequacy of the range of responses to be recorded.
8. Other	Look for problems not identified in Steps 1–7.

Following decisions related to retention, deletion, and revision of items, we will review the new PALCARE tool to ensure its items reflect all eight domains of PC listed in the NCP guidelines and the major dimension of Readiness to Change Framework.

This study is a three-years project. The timeline and study related activities are listed in Table 4.

**Table 4:** Timeline for Study Related Activities.

Tasks	Pre-award	Year 1/Quarter				Year 2/Quarter				Year 3/Quarter			
		**1**	**2**	**3**	**4**	**1**	**2**	**3**	**4**	**1**	**2**	**3**	**4**
Obtain IRB approval													
Start up													
Hire and train research assistants													
Aim 1: Phase 1 Semi-structured interview; data analysis & initial items for PALCARE tool													
Aim 2: Phase 2 Two rounds of expert panel review and analysis													
Aim 2: Phase 3 Focus group with experts and analysis													
Aim 2: Phase 4 Cognitive interviews with dyads and analysis													
Aim 3: Phase 4 Convergent validity and test-retest reliability													
Disseminate findings and prepare future proposal													
Close out													

## Discussion

Informed by the NCP project guidelines, readiness to change framework, and principles of community-engaged research, this 3-year R15 research study will iteratively develop and establish the content validity, preliminary convergent validity, and test-retest reliability of a palliative care readiness tool for older adults with cancer. To our knowledge the PALCARE would be the first standardized tool to assess readiness for early PC in older adults with cancer. Once the reliability and validity of the PALCARE tool has been established, it can be used clinically by the primary oncology team to assess readiness for PC and refer older adults with cancer to appropriate PC services. The utility of this tool is threefold: a) assess readiness for early PC in older adults with cancer, b) provide/deliver targeted PC that is congruent with their readiness level in the domains where they feel ready, and c) design tailored interventions to inform, educate, and support those who are reluctant or less ready to consider PC in general or do not feel ready yet in certain domains of PC. Over time, this may help facilitate readiness to accept early PC referral and ultimately positive health outcomes and improved quality of life. This is especially important for older adults with cancer who have lower early PC use, including those who belong to racial minority groups, low socio-economic status, or reside in rural under-resourced areas. This tool could also influence tele-PC interventions aimed at underrepresented patients in rural or under-resourced areas. Future applications can be expanded to all adult oncology patients, family caregivers, and other seriously ill populations. Consistent with the goal of an R15 study, additional impact of this project includes planned activities to enhance the university’s research environment by providing opportunities for students with limited to no prior interdisciplinary clinical research experience with rich research opportunities.

## Future directions

After completing this study and developing the PALCARE tool, our next step is to establish the psychometric validity and reliability of the PALCARE tool by conducting quantitative studies. After we establish the validities and reliabilities of the PALCARE tool, our longer-term goal will be to conduct a randomized controlled trial to examine if the use of a PALCARE tool will result in early PC referral and utilization among older adults with cancer leading to an improved QoL.

## Dissemination plans

Study results will be disseminated using multiple platforms included but not limited to a) peer-reviewed journal article publications in multiple leading journals such as *Journal of Geriatric Oncology, Journal of Hospice and Palliative Nursing, Journal of Palliative Medicine, American Journal of Hospice and Palliative Medicine* b) presentations at annual conferences of several national and international geriatric, palliative care, oncology, and nursing professional organizations such as the Gerontological Society of America (GSA), International Society of Geriatric Oncology (SIOG), Hospice and Palliative Nurses Association, American Academy of Hospice and Palliative Medicine (AAHPM), Southern Nursing Research Society (SNRS) c) social media platforms such Blue Sky, Twitter, and LinkedIn, and d) community engagement activities such as clinical site events, stakeholder meetings or community forums.

Once the results have been disseminated via publications and conference presentations, the de-identified data sets will be made available to interested researchers. Public use and restricted access study data and associated documentation will be made available to the research community free of charge through the deposit at National Archive of Computerized Data on Aging (NACDA).

## Conclusion

In conclusion, this 3-year research project will iteratively develop and validate a palliative care readiness tool which is critically essential for older adults with cancer who have high symptom burden and unmet needs but are highly likely to underutilize palliative care. Our PALCARE tool has great potential to reduce existing disparities and promote equitable access and utilization of palliative care among older adults with cancer.
